# Large Scale Replication Study of the Association between HLA Class II/*BTNL2* Variants and Osteoarthritis of the Knee in European-Descent Populations

**DOI:** 10.1371/journal.pone.0023371

**Published:** 2011-08-10

**Authors:** Ana M. Valdes, Unnur Styrkarsdottir, Michael Doherty, David L. Morris, Massimo Mangino, Agu Tamm, Sally A. Doherty, Kalle Kisand, Irina Kerna, Ann Tamm, Margaret Wheeler, Rose A. Maciewicz, Weiya Zhang, Kenneth R. Muir, Elaine M. Dennison, Deborah J. Hart, Sarah Metrustry, Ingileif Jonsdottir, Gudbjorn F. Jonsson, Helgi Jonsson, Thorvaldur Ingvarsson, Cyrus Cooper, Timothy J. Vyse, Tim D. Spector, Kari Stefansson, Nigel K. Arden

**Affiliations:** 1 Department of Twin Research and Genetic Epidemiology, King's College London, London, United Kingdom; 2 deCODE Genetics, Reykjavik, Iceland; 3 Academic Rheumatology, University of Nottingham, Nottingham, United Kingdom; 4 Divisions of Genetics and Molecular Medicine and Immunology, Infection and Inflammatory Diseases, King's College London, London, United Kingdom; 5 Department of Internal Medicine, University of Tartu, Tartu, Estonia; 6 Department of Sports Medicine and Rehabilitation, University of Tartu, Tartu, Estonia; 7 Respiratory & Inflammation Research Area, AstraZeneca, Loughborough, United Kingdom; 8 Health Sciences Research Institute, Warwick Medical School University of Warwick, Coventry, United Kingdom; 9 Medical Research Council Lifecourse Epidemiology Unit University of Southampton Southampton, United Kingdom; 10 Faculty of Medicine, University of Iceland, Reykjavik, Iceland; 11 Department of Medicine, Landspitali University Hospital, Reykjavik, Iceland; 12 University Hospital, Institution of Health Science, University of Akureyri, Akureyri, Iceland; 13 Musculoskeletal Biomedical Research Unit, University of Oxford, Oxford, United Kingdom; Marienhospital Herne - University of Bochum, Germany

## Abstract

Osteoarthritis (OA) is the most common form of arthritis and a major cause of disability. This study evaluates the association in Caucasian populations of two single nucleotide polymorphisms (SNPs) mapping to the Human Leukocyte Antigen (HLA) region and deriving from a genome wide association scan (GWAS) of knee OA in Japanese populations. The frequencies for rs10947262 were compared in 36,408 controls and 5,749 knee OA cases from European-descent populations. rs7775228 was tested in 32,823 controls and 1,837 knee OA cases of European descent. The risk (major) allele at rs10947262 in Caucasian samples was not significantly associated with an odds ratio (OR)  = 1.07 (95%CI 0.94 -1.21; p = 0.28). For rs7775228 the meta-analysis resulted in OR = 0.94 (95%CI 0.81-1.09; p = 0.42) for the allele associated with risk in the Japanese GWAS. In Japanese individuals these two SNPs are in strong linkage disequilibrium (LD) (r^2^ = 0.86) with the HLA class II haplotype DRB1*1502 DQA1*0103 DQB1*0601 (frequency 8%). In Caucasian and Chinese samples, using imputed data, these SNPs appear not to be in LD with that haplotype (r^2^<0.07). The rs10947262 and rs7775228 variants are not associated with risk of knee OA in European descent populations and they do not appear tag the same HLA class II haplotype as they do in Japanese individuals.

## Introduction

Osteoarthritis (OA) is the most common form of arthritis and the leading cause of physical disability among the elderly in industrialized nations with severely impaired quality of life due to pain and loss of joint functioning [1]. Knee OA is a complex disease with a multi-factorial etiology which includes both genetic and environmental factors [2]. Although OA is not an inflammatory arthropathy, inflammation-related factors have been shown to be implicated in the pathogenesis of OA [3]. A number of studies have been carried out to determine the genetic determinants of knee OA and a large scale Japanese genome-wide association scan (GWAS) has identified two single nucleotide polymorphisms (SNPs) mapping to the human leukocyte antigen (HLA) gene region to be strongly associated with knee OA [4]. One of the markers identified, rs7775228, mapped to the HLA class II gene *DQB1* and reached genomewide significant in Japanese samples (OR = 1.34 95% CI 1.21–1.49 p = 2.43×10^−8^) but showed no evidence of association in combined European populations used for replication in the same study (OR = 0.93 95%CI 0.76–1.13). The other marker identified mapped to intron 1 of the butyrophilin-like 2 gene (*BTNL2*) gene which has been implicated in T-cell activation [4]. The major allele at rs10947262 was significantly associated with increased risk of knee OA in the Japanese discovery and replication samples combined (OR = 1.32 95%CI 1.19–1.46; p = 6.7×10^−8^). In the Caucasian samples in that study this marker appeared to be associated in the same direction (OR = 1.29 95%CI 1.03–1.61 p = 0.025) and combining both Japanese and Caucasian data rs10947262 achieved genomewide significance (defined as p≤5×10^−8^) [5] overall. A subsequent study by Shi and co-workers tested the same two SNPs in a Chinese case-control study and an Australian case-control study but failed to find a significant association for these SNPs in either group [6].

The proteins encoded by the classical HLA class I and class II genes are highly polymorphic, have been implicated in the pathogenesis of over 100 diseases, including arthropathies like rheumatoid arthritis and systemic lupus erythematosus and play an essential role in self/non-self immune recognition [7]. The presence of activated T cells and Th1 cytokine transcripts in chronic joint lesions of patients with OA suggest that T cells contribute to chronic inflammation [8], [9]. T cells and chondrocytes have been reported to interact through cell surface molecules such as HLA, CD4 or CD8 in OA [8] and proliferative responses of peripheral blood T cells from patients with OA are substantially higher than those of control T cells from normal donors [9]. These observations support a possible role for HLA variation in genetic susceptibility to OA in agreement with the results from the Japanese GWAS. Therefore, we have aimed to investigate whether we could confirm the role of the two variants identified in Japanese and mapping to these important immune response molecules in risk of knee OA in European descent populations.

## Methods

### Study cohorts

All the study subjects provided written informed consent to participate. This research project was approved by the ethical committees at all participating institutions as follows: The deCODE case-control study protocol was approved by the Data Protection Authority of Iceland and the National Bioethics Committee of Iceland. The Nottingham case-control and the GOAL study protocols were approved by the research ethics committees of Nottingham City Hospital and North Nottinghamshire, UK. The Chingford study protocol was approved by the Guys & St Thomas' Trust and Waltham Forest Trust ethics committees approved the study protocol. The TwinsUK study protocol was approved by St Thomas' Hospital Research Ethics Committee. The Hertfordshire Cohort Study protocol approval was obtained from East and North Hertfordshire Ethics Committee. The Estonian Study protocol was approved by the Ethics Committee of the University of Tartu.

#### 1- deCODE case-control

A list of patients with OA of the hand, knee and hip was obtained on the basis of patients' records at hospitals and health care centers in Iceland [10]. Controls were individuals with no external signs of OA in any joint who did not have a diagnosis of primary OA. For the present study there were 1068 knee OA cases, and 31,654 controls included.

#### 2- Nottingham case-control

All cases had been referred to hospital with symptomatic, clinically severe knee OA and the majority had undergone unilateral or bilateral total knee replacement (TKR) within the previous 5 years. Patients (aged 40–90) were included if they had a diagnostic code of primary OA. Height and weight were measured to calculate BMI. Pre-operative knee radiographs [11] were scored for individual radiographic features of OA by a single observer and graded 0–3 according to a standard atlas using the Kellgren and Lawrence (K/L) grade for the tibiofemoral compartment of each knee [12]. Self-reported ethnicity was assessed by a nurse administered questionnaire and only individuals of European descent were included in the genetic study. Controls were unaffected sibs of joint replacement probands, free from radiographic OA and over the age of 45. Subjects aged 45–85 who had undergone intravenous urography (IVU) in the same hospital were assumed to represent the average genetic susceptibility of the general population and recruited as unrelated controls. Further details are described elsewhere [13].

#### 3- Genetics of Osteoarthritis and Lifestyle (GOAL) study

Recruitment criteria were the same as for the Nottingham case-control study. Cases with clinically severe knee OA were recruited from hospital orthopaedic surgery total knee replacement (TKR) lists in the Nottingham area as previously described [14]. Approval for recruitment was obtained from the research ethics committees of Nottingham City Hospital and North Nottinghamshire. Knee radiographs were examined to confirm the diagnosis and to grade for changes of OA and scored for individual radiographic features of OA by a single observer and graded 0–3 according to a standard atlas using the K/L grade for each knee joint [12].Only individuals of European descent were included in the genetic study. Subjects aged 45–85 who had undergone intravenous urography (IVU) in the same hospital were recruited as controls and underwent clinical examination and radiographs of the pelvis and both knees.

#### 4- The Chingford Study

A prospective population-based longitudinal cohort of women of European descent (assessed by questionnaire) derived from the register of a large general practice in North London, representative of the general UK population. The study design and rationale are described elsewhere in detail [15]. The K/L grade was scored for the tibiofemoral compartment of each knee [12]. Controls were individuals from the population with no knee OA.

#### 5- The TwinsUK study cohort

A case-control substudy of OA was derived from the TwinsUK Adult Twin Registry which is a population-based sample of twins from the UK studying the hereditary basis of a wide variety of age-related traits and diseases (http:/www.twinsUK.ac.uk). These twins were recruited from the general population. Only women participated in an X-ray study to investigate the heritability of radiographic OA. From those participating in such study, 348 unrelated Caucasian women (only one twin from each pair) aged 55 at the time of the visit with both a knee and a hip K/L<2 at both the femoro-acetabular and tibio-femoral compartments were selected as controls for genotyping the present study. Cases were unrelated Caucasian women from the same cohort with severe knee OA who had undergone unilateral or bilateral or total knee replacement (n = 43). All subjects included in this analysis were unrelated so that no adjustment for relatedness was required.

#### 6- The Hertfordshire Cohort Study

A large population-based study. Details of the study design have been published previously [16]. Ethical approval was obtained from East and North Hertfordshire ethical committees and all participants gave written informed consent. Weight bearing anteroposterior and lateral semi-flexed radiographs of both knees were. Radiographs were graded at the tibio-femoral and patello-femoral joints using a standard atlas and the K/L score was determined [20]. A K/L grade ≥2 was defined as definite OA. For consistency with the other radiographic cohorts in this study, only data on the tibiofemoral compartment has been included here.

#### 7- The Estonian Study

A population based sample from the Estonian towns of Elva and Võru (421 subjects), aged 32–55 years was recruited using primary care lists [17]. In addition 95 consecutive arthroscopy patients, with the same age range, were recruited from the clinic. All subjects had standardised weight-bearing antero-posterior radiographs of the tibiofemoral joint. Joint space narrowing (JSN) and presence of osteophytes, were graded independently by two radiologists using a grading system (grades 0 –III) based on a line-drawing atlas [18]. The JSN and osteophytes grades were then used it to derive K/L score. Individuals with a K/L grade ≥2 were considered cases, otherwise were classified as controls.

### Genotyping

#### deCODE

The rs7775228 SNP was assayed with various Illumina Bead Chips ( Infinium HumanHap300, HumanHap300-Duo, humanCNV370-Duo, Human610-Quad, Human1M or Human1M-Duo SNP chips) excluding all samples with a yield <98% from the analysis. The SNP had a call rate of over 97% on each of the chip types, a combined call rate of over 98% and did not deviate from Hardy Weinberg equilibrium on any chip type. The rs10947262 SNP was assayed with the Human1M or Human1M-Duo BeadChips (Illumina) on 1,148 individuals. Genotypes into the 32,000 samples assayed with the other chip types and used in this study were imputed into pre-phased samples based on Icelandic training set as described in [19]. The imputation method uses the same statistical model as that used in the IMPUTE software [20]. The agreement between directly genotyped genotypes and imputed genotypes was 99%.

Similar methods were applied to the public HLA and SNP dataset in [21] to impute the most likely genotypes for rs10947262.

Genotyping in the samples from Hertforshire, Chingford, Nottingham (GOAL), TwinsUK and Estonia was carried out by Kbioscience Ltd, Hertfordshire UK. SNPs were genotyped using the KASPar chemistry, which is a competitive allele-specific PCR SNP genotyping system using FRET quencher cassette oligos (http://www.kbioscience.co.uk/genotyping/genotyping-chemistry.htm).

### Statistical Analysis

Allelic odds ratios (OR) were calculated by comparing the frequencies among cases and controls and the p-value was computed using a Pearson's chi-square test. In the absence of inter-study heterogeneity we constructed a Mantel-Haenszel meta-analysis of data from the samples to assess the overall evidence of association. The assumption of heterogeneity for each analysis was tested using the DerSimonian-Laird method [22] and heterogeneity was evaluated with Q-statistic and I^2^. Random effects models were used only if evidence of heterogeneity was observed (I^2^>20%). The R software version 2.10.1 was used (The R Foundation for Statistical Computing http://www.r-project.org/). Linkage disequilibrium was computed using Haploview v 4.2 (http://www.broadinstitute.org/haploview).

## Results

### Genetic Association Study

The characteristics of the data used for replication and the allele frequencies at the SNPs under study are shown in **Table 1**. As is common for polymorphisms within the HLA region we see considerable variation within European populations [7].

**Table 1:** Characteristics of cases and controls of unpublished studies included in the analysis.

Study cohort		n	Age yrsmean (SD)	BMI kg/m^2^ mean (SD)	Females (%)	Country of origin	MAF%rs7775228 *^(1)^*	MAF% rs10947262 *^(2)^*
deCODE	kneeOA	764	70.0 (7.3)	28.9 (4.9)	63.9%	Iceland	10.9%	9.2%
	controls	31,237	59.3 (20.7)	27.0 (5.3)	54.5%		9.8%	10.0%
Nottingham	kneeOA	1,831	68.7 (8.9)	29.7 (5.4)	55.3%	UK	N/A	6.7%
	controls	729	66.3 (7.4)	26.6(3.9)	57.4%			6.6%
GOAL	kneeOA	1,584	68.5 (7.1)	30.7 (5.4)	46.9%	UK	N/A	6.4%
	controls	745	62.6 (8.4)	27.1 (4.4)	49.9%			6.5%
Chingford	kneeOA	254	66.4 (6.4)	28.4 (5.3)	100%	UK	N/A	4.9%
	controls	531	63.2 (5.9)	26.2 (4.2)	100%			7.2%
TwinsUK	kneeOA	41	65.9 (9.9)	27.1 (3.2)	100%	UK	N/A	9.8%
	controls	349	67.6 (5.6)	25.2 (4.2)	100%			6.0%
Hertfodshire	kneeOA	146	65.2 (2.6)	29.5 (5.1)	41.2%	UK	N/A	5.5%
	controls	776	64.8 (2.7)	26.2 (3.8)	50.1%			6.8%
Estonia	kneeOA	68	51.0 (6.0)	30.6 (5.7)	65.7%	Estonia	N/A	12.5%
	controls	447	46.5 (6.2)	27.7 (5.2)	69.8%			8.9%

MAF =  minor allele frequency.

(1) **Minor allele = C, major allele = T.**

(2) **Minor allele = T, major allele = C.**

**Figure 1:**
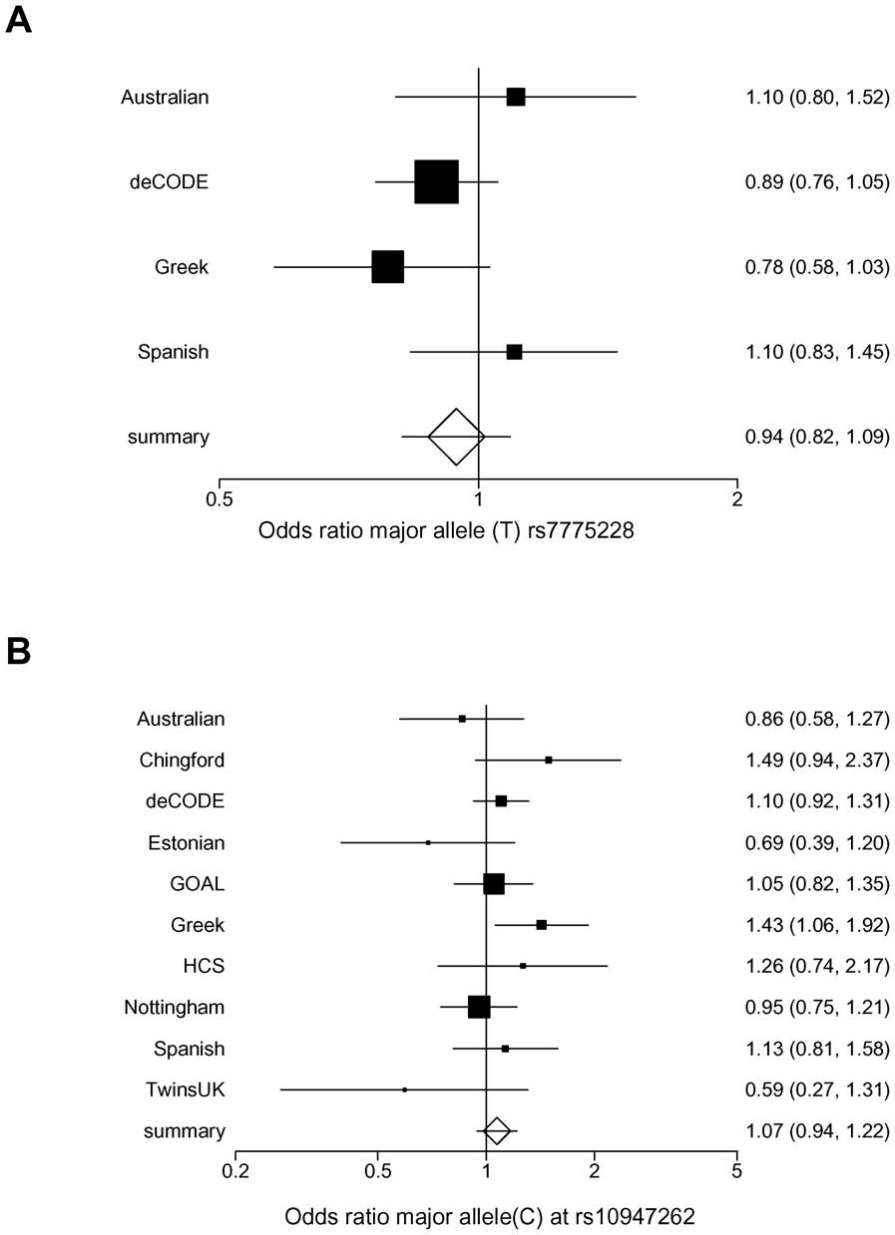
Meta-analysis of genetic association between two HLA SNPs and knee OA. Forest plot of study-specific estimates and random-effects summary effect size and 95% confidence intervals (95%CIs) for the genetic association with knee OA of (**A**) the major allele (T) at rs7775228 in three published studies and the deCODE case-control study (**B**) the major allele (C) at rs10947262 in three published studies and seven additional European studies. Square sizes are proportional to the number of cases in each study.

We found no evidence for association with rs7775228 in the Icelandic cohort nor overall combining data with the published Australian [6], Spanish and Greek data [4] (**figure 1A**). The overall odds ratio (OR) for the risk (major) allele in Caucasians is 0.94 (95%CI 0.81-1.09; p = 0.42; I^2^ = 26%), using a random-effects model due to the value of heterogeneity being higher than 25%. Similar results were obtained however using a fixed-effects meta-analysis (OR = 0.94; 95%CI 0.81 -1.09; p = 0.27). The confidence intervals for the odds ratio in combined Caucasian samples do not overlap the confidence intervals in Japanese for this SNP (OR = 1.34 95% CI 1.21–1.49) and the effect of this SNP in risk of knee OA is significantly different with p<1.2×10^−4^ between these two ethnic groups.

For rs10947262, which was originally reported to be associated in combined Spanish and Greek samples in the same direction as in Japanese cases, we also find overall no evidence of association OR = 1.07 (95%CI 0.94 -1.21; p = 0.28) for the allele associated with risk in the initial study using a random effects model (I^2^ = 27% p (Cochran's Q) <0.19) although in this case the odds ratios in Japanese (OR = 1.32 95%CI 1.19–1.46) and Caucasians are overlapping.

Unlike rs7775228 where all genotypes were directly typed, the genotypes for rs10947262 for the largest dataset (deCODE) were imputed. Therefore we assessed whether the use of an imputed dataset might be influencing our conclusions. Excluding the imputed dataset we confirmed that there was no association between this marker and knee OA having obtained an OR = 1.06 (95%CI 0.91 -1.24; p = 0.45) for the allele associated with risk in the initial study using a random effects model (I^2^ = 37% p (Cochran's Q) <0.12).

We hypothesized that the initial association reported by the Japanese group [4] might be due to a role of the *BTLN2* locus itself, represented by these two SNPs, in risk of OA. The statistical power available in our study – including both published and new data - to detect an association between rs7775228 and knee OA was 80% for an odds ratio of 1.17 for a significance level α = 0.05. For rs10947262 an OR≥1.12 is needed for α = 0.05. Thus, although it is widely known that initial reports tend to overestimate genetic effect sizes, our study is powered to detect effects considerably smaller than the initial report and for genomewide significance, so much so as to be below the lower 95% CI from the initial report in [4]. Given the lack of replication in Caucasians of this association the hypothesis of a direct role of either of these SNPs in risk of knee OA seems unlikely.

Alternatively, the genetic association detected might have been due to linkage disequilibrium (LD) between rs7775228 and rs10947262 and classical HLA loci and the lack of replication might be explained by differences in the pattern of LD across ethnic groups, which in the case of HLA are well documented to vary extensively even between ethnically similar populations (e.g. Asian/Pacific populations [23]). We have therefore investigated if the differences observed could be explained by differences in LD between the initial population studied (Japanese) and the subsequent replication samples of Chinese and Caucasian descent.

### Linkage disequilibrium with classical HLA class II loci

De Bakker and co-workers [21] determined LD patterns between the highly polymorphic HLA genes and background variation by typing the classical HLA genes and >7,500 common SNPs and deletion/insertion polymorphisms across four population samples, including European descent and Japanese samples. That analysis provided informative tag SNPs to capture some of the variation in the MHC region and is publicly available online (http://www.inflammgen.org/index.php?option=com_content&task=view&id=25&Itemid=42). We used this database to investigate which classical HLA genes are in LD with the two SNPs identified by the Japanese study.

Among the Japanese samples included in this public domain resource the minor allele at rs7775228 tags the HLA class II haplotype DQA1-0103-DQB1*0601 which has a frequency of 17.05% in Japanese (r^2^ = 0.38). Although rs10947262 was not directly genotyped in the study by De Bakker et al [21], the large amount of closely markers available in this set made it possible for us to apply standard imputation methods [20] to impute the genotypes for rs10947262 in this dataset for all three ethnic groups under study.

**Figure 2:**
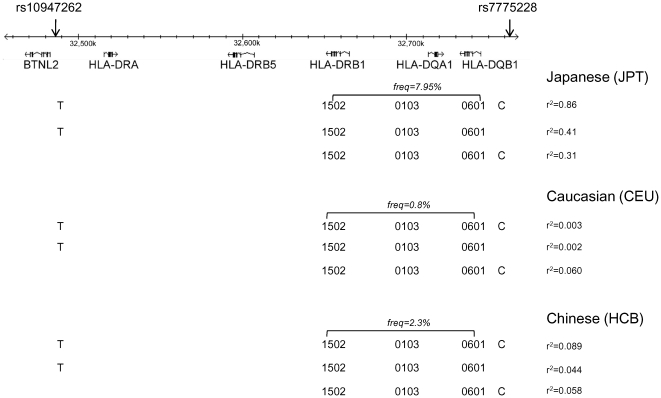
Map of the HLA class II region in harboring markers rs7775228 and rs10947262. The frequency of haplotype DRB1*1502 DQA1*0103 DQB1*0601 and its linkage disequilibrium with rs7775228 and rs10947262 in three ethnic groups is shown.

In Japanese samples the minor allele at rs10947262 is in positive LD with DRB1*1502 with r^2^ = 0.512. DRB1*1502 has a frequency of 10.05% in Japanese samples and is itself in LD with DQA1-0103-DQB1*0601 (r^2^ = 0.27) in this ethnic group. The haplotype formed by the minor alleles at rs7775228 and rs10947262 (haplotype frequency 8.05%), which was found to be more strongly associated in Japanese cases than either individual marker, tags haplotype DRB1*1502 DQA1*0103 DQB1*0601 with r^2^ = 0.864 (**Figure 2**). Therefore, these two SNPs are tagging a specific class II antigen presenting haplotype.

The DRB1*1502 DQA1*0103 DQB1*0601 HLA class II haplotype is present in Caucasian samples in the public domain HLA-SNP map set [21] in 3 out of 360 haplotypes (frequency = 0.8%). The LD with the minor allele at rs7775228 is r^2^ = 0.060 and with rs10947262 is only r^2^ = 0.002. The haplotype formed by the minor alleles at these two loci has an r^2^ = 0.003 with DRB1*1502 DQA1*0103 DQB1*0601 (**Figure 2**). We further confirmed that the frequency of the DRB1*1502 DQA1*0103 DQB1*0601 in Caucasians is very low in the literature in a large study of Caucasian samples (derived from 606 pedigrees, 2424 individuals) where this haplotype was reported to have a frequency of 0.7% [24].

In Han Chinese from Beijing (HCB) samples in the De Bakker study [21] DRB1*1502 DQA1*0103 DQB1*0601 has a frequency of 2.3% and the LD with rs7775228 is r^2^ = 0.058, r^2^ = 0.044 with rs10947262 and r^2^ = 0.090 with the haplotype formed by the minor alleles at both SNPs. (**Figure 2**). Therefore, in Chinese and Caucasian samples these two SNPs are not tagging the HLA DRB1-DQA1-DQB1 haplotype that they tag in Japanese samples.

## Discussion

In this study we failed to find an association between markers rs7775228 and rs10947262 and knee OA in European descent samples in spite of a sample size large enough to have sufficient statistical power to confirm the effect sizes previously reported and even much more modest genetic effects. We could not replicate in European descent samples the findings of Nakajima and co-workers [4] who identified a genome-wide significant association between these two SNPs and knee OA in Japanese subjects. Furthermore, Shi et al [6] were unable to find an association in Chinese OA patients for these same SNPs.

There have been several studies analyzing genomewide significant variants directly across ethnicities with varying results [25]–[26]. Because of differences in underlying LD structure and variant frequency between ancestral populations the tagging relationship between a SNP and a pathogenic variant may be disrupted. Therefore a non-replication of a particular variant must be considered inconclusive and warrant further investigation as demonstrated in a recent study of breast cancer at the *ESR*1 locus [27]. Having analyzed the LD between the two SNPs ( rs7775228 and rs10947262) identified by GWAS observation in the Japanese study by Nakajima and co-workers [4] and the classical loci in the HLA class II region, we can infer that the DRB1*1502 DQA1*0103 DQB1*0601 haplotype is protective for risk of OA in Japanese samples. Thus, association of these two SNPs with OA in the Japanese samples may be due to their property of tagging this haplotype. The frequency of this HLA haplotype varies between ethnic groups; it is present in 8% frequency in Japanese samples, 2.3% in Chinese samples and is only in 0.7–0.8% frequency in European samples. Moreover, the two SNPs are not in LD with this haplotype in European or in Chinese samples, possibly explaining the non-replication of these SNPs. A suitable tag SNP for this particular DRB1-DQB1 haplotype in European samples is rs2858880 [21] which has r^2^∼1.0 with DRB1*1502 DQA1*0103 DQB1*0601. The minor allele at this SNP is very rare and to achieve 80% power and p<0.05 even assuming a strong protective effect (OR = 0.7, i.e. non carriage of this haplotype results in OR = 1.4) it would require 10,586 knee OA cases and 10,586 controls, i.e., almost twice as many cases as were tested in our study. Ideally, however, the HLA loci should be genotyped directly (e.g.using PCR-based sequence specific oligonucleotide probe methods) in order to achieve conclusive validation results and to assess the role of any other DRB1-DQA1-DQB1 haplotypes in risk of OA.

We note that there are some potential study limitations, in particular the different definitions of knee OA used by the various studies, some of which focus on total knee replacement as an outcome and others that have focused on radiographic features and for OA phenotypic definitions which reflect a different subset of OA have been shown to influence the ability to detect genetic associations [28]. The initial discovery was made in Japanese samples using a definition of radiographic OA (K/L≥2) and pain [4]. The association with rs10947262 initially was replicated in a Greek study where the outcome as total knee replacement (TKR) but the association with rs7775228 was not replicated in that same cohort [4]. In fact, rs7775228, the Icelandic, Spanish and Greek studies all used TKR as the outcome and show no evidence of association. For rs10947262 we cannot distinguish a clearly different pattern for studies where only a radiographic definition was used (e.g. Chingford, TwinsUK, HCS, Estonia) from those where symptoms or even TKR were part of the recruitment criterion (e.g. Nottingham, GOAL, deCODE). Further, for knee OA it has been shown in a case where the functional variant of a gene is truly involved in risk of disease (the promoter variant in the *GDF-5* gene) that, these differences in phenotype definition do not deter from observing an association in Caucasians with genome-wide statistical significance even if it had been originally discovered in Asian samples [29].

Another study limitation to our conclusions is that the data used to derive inferences concerning LD with HLA haplotypes comes in part from imputed data. Therefore, although imputation for this SNP appears to be reliable, it is unlikely but possible that the conclusions might have been different using directly typed data.

Neither our results nor those from Shi et al [6] preclude an important role for variation in the HLA region in genetic susceptibility to OA. It is indeed possible that class II antigen presenting molecules may be involved in the pathogenesis of OA. In order to determine if DRB1 -DQA1-DQB1 haplotypes (and antigen presenting molecules in general), are involved in risk of knee OA in Caucasian populations, further studies which take into account the complex LD structure and the extremely high genetic diversity of this region are needed.
